# Standardization and Validation of a Methodology for Assessing Least Reflection Factor and Skin Lipid Replenishment Using Skin Reflectance and Biophysical Measurements

**DOI:** 10.7759/cureus.110086

**Published:** 2026-06-02

**Authors:** Maheshvari N Patel, Nayan Patel, Apeksha Merja

**Affiliations:** 1 Clinical Research, NovoBliss Research Private Limited, Ahmedabad, IND; 2 Pharmacology, Swaminarayan University, Ahmedabad, IND; 3 Clinical Research Operations, NovoBliss Research Private Limited, Ahmedabad, IND; 4 Dermatology, NovoBliss Research Private Limited, Ahmedabad, IND

**Keywords:** least-reflection factor, skin lipid replenishment, skin reflection, skin sebum, skin shine

## Abstract

Background: Assessment of skin lipid replenishment and surface appearance requires standardized methodologies capable of measuring both skin-reflecting and barrier-related skin properties. The least-reflection factor (LRF) reflects skin shine and surface lipid presence and is influenced by skin shine and sebum levels. Furthermore, lipid replenishment of the stratum corneum is associated with barrier-related parameters such as skin hydration, transepidermal water loss (TEWL), and surface scaliness. Integrating the reflecting and biophysical parameters may provide a comprehensive approach for evaluating skin surface refinement and barrier restoration; however, standardized methodologies combining these parameters remain limited.

Objective: To standardize and validate a reliable and reproducible methodological framework for assessing the LRF and skin lipid replenishment using biophysical measurements, focusing on skin shine, sebum levels, hydration, TEWL, and skin surface microrelief parameters.

Methods: This single-center, exploratory, validation study used an open-label, split-face design, including fifteen healthy adult participants with normal to dry and sensitive skin. Each participant served as their own control, with a lipid-replenishing topical product applied to one facial site and water applied to the opposite site. Assessments included skin shine and LRF, sebum levels, skin hydration, TEWL, and skin surface microrelief parameters such as smoothness, roughness, and scaliness. Measurements were performed at baseline and 15 minutes after application under controlled environmental conditions.

Results: Post-application evaluations at the test site demonstrated increased skin hydration by 46.20 ± 5.87 (p value <0.0001), reduced TEWL by 8.26 ± 0.63 (p value <0.0001), and improved surface smoothness by 361.70 ± 63.99 (p value <0.0001), skin shine by 4.14 ± 0.12 (p value <0.001), reduced skin sebum level by 11.99 ± 3.68 (p value <0.01) while control sites remained within physiological ranges. Skin surface shine remained unchanged, and sebum levels showed no variation, indicating that changes in the LRF were directionally consistent.

Conclusion: This study established a standardized methodology for evaluating LRF and lipid replenishment-related skin changes using integrated skin reflectance and biophysical measurements. The methodology demonstrated the ability to detect instant changes in skin barrier function and surface morphology. LRF showed potential as a reflecting parameter associated with skin shine and surface characteristics, supporting its use in dermatological and cosmetic research evaluating skin surface quality and barrier restoration.

## Introduction

The integrity of the skin barrier is fundamentally governed by the organization and composition of lipids within the skin stratum corneum. These lipids are important for keeping epidermal cohesion; maintaining transepidermal water flow through the skin, protecting surface smoothness; and making sure that the skin's reflecting properties, such as skin reflection factor measured through skin shine, are the same all over. Damage to the intercellular lipid matrix compromises barrier function, causing more transepidermal water loss (TEWL) [1-3].

As dermatological and cosmetic research-based evidence becomes more emphasized, there is a greater need for methodologies that are capable of detecting small changes associated with skin lipid replenishment. Traditional visual grading and subjective evaluations are fundamentally constrained by inter- and intra-observer variability and frequently lack the requisite sensitivity required to detect early or modest changes in skin surface characteristics [4]. As a result, methodologies measuring skin hydration, skin shine, TEWL, skin surface microrelief, sebum balance, and reflection have become essential tools in recent skin research for evaluating products [5-7].

Even though each methodology gives useful isolated measurements, lipid replenishment is a complex process that shows up through interconnected changes in surface morphology, hydration change, and TEWL. The reflectance of the skin's surface is affected not only by water content but also by lipid arrangements and skin micro-surface smoothness. Better lipid arrangement in the skin surface reduces surface irregularities, enhances bonds, and promotes more uniform reflection of light, whereas reduced concentration of lipids or rough skin scatters light diffusely, resulting in less surface brightness and shine [8-10].

The least-reflection factor (LRF), derived from the ratio of directly reflected (specular) light to diffusely scattered light, represents a composite optical parameter reflecting skin surface characteristics. This parameter is obtained using non-invasive optical measurements and is influenced by multiple factors, including surface smoothness, hydration status, and sebum distribution. Variations in these properties alter the balance between specular and diffuse reflection, thereby modulating overall skin reflectance behavior.

Unlike single-parameter endpoints, LRF is intended as an exploratory integrated indicator that captures combined surface changes associated with lipid replenishment and barrier-related improvement. Previous studies have demonstrated relationships between surface roughness, hydration, and optical reflectance properties [11-13], supporting the rationale for composite optical assessment. However, the application of such parameters requires careful standardization and validation in relation to established biophysical measurements, which form the basis of the present study.

Despite the availability of sophisticated skin assessment tool, there still remains a need for standardization and methodological frameworks that reflects the ability of measuring of skin lipid replenishment changes. Validation studies of methodology, using standardized environmental parameters, intra-subject comparison designs, and controlled application procedures, are essential to ensure that the results of the instrumentation are scientifically valid and can be generalized to future studies [14,15].

The current study was therefore planned as a standardization and validation study plan to evaluate a set of skin measurement methods for their ability to detect changes connected with lipid replenishment and reflection factor. By examining the responsiveness and relationship between these parameters, the current study aims to provide a methodological framework that could be used as a basis for future dermatological and cosmetic research on skin barrier repair and lipid replenishment.

The main objective of the current study was to standardize and validate methodology for assessing lipid replenishment-related changes in skin parameters, including TEWL, hydration, skin surface microrelief (smoothness, roughness, and scaliness), and LRF-related changes in skin sebum levels and skin shine. The main emphasis was focused on evaluating the LRF as an integrated skin-reflecting marker of surface refinement and barrier-related improvement in controlled experimental conditions.

## Materials and methods

Study design

This standardization and validation study was designed as an open-label, split-face, single-day study conducted to standardize and validate methodology for the assessment of changes in lipid replenishment in skin reflectance and biophysical skin parameters.

The study was performed at NovoBliss Research Private Limited (Ahmedabad, India) in accordance with the ethical standards described in the Declaration of Helsinki, the International Conference on Harmonisation-Good Clinical Practice (ICH-GCP) guidelines, and the ethical standards of the Indian Council of Medical Research (ICMR). The study was prospectively registered with the Clinical Trial Registry of India (CTRI No.: CTRI/2026/01/102475) and ClinicalTrials.gov (Identifier: NCT07423325). The first participant’s first visit and the last participant’s last visit occurred on January 28, 2026.

Ethical approval and informed consent

The study validation plan was reviewed and approved by the ACEAS' Independent Ethics Committee before study initiation on January 17, 2026, with the Identifier number NB250045-NB-V. Written informed consent was obtained from all participants before the initiation of any study-related procedures. The participants were previously informed about the study objectives, procedures, potential risks, and their right to withdraw from the study at any time without penalty.

Study population

Fifteen (15) healthy adult male and female participants aged between 18 and 55 years were enrolled in this exploratory standardization and validation study.

Eligibility criteria

Subjects with normal to dry skin and in generally good health at the time of enrollment were included in the study. Written informed consent was obtained from all participants prior to participation in the study.

Participants were asked to avoid using any other skincare and cosmetics products on the face during the entire study period and mandatory not have participated in any other similar clinical or cosmetic investigation study within the previous three months.

Participants were not eligible if they had any active dermatological condition or history of chronic skin disease that could affect the assessments in the study. Other reasons for exclusion included known hypersensitivity or allergic reactions to cosmetic products; use of topical dermatological medications in the past four weeks or systemic medications within three months previous to enrollment; pregnancy and lactation; substance abuse; history of chronic systemic illness affecting skin physiology; and participation in other cosmetic, device, or therapeutic studies within four weeks prior to study initiation. Participants taking chronic medications that could affect skin barrier function or instrumental assessments were also not eligible at the discretion of the investigator.

Study procedure

A split-face, single-visit study design was used, with each participant acting as their own control to minimize inter-subject variability. One facial site was assigned as the test site and the opposite facial site assigned as the control site.

Instrumental evaluations were made on both facial sites before any product was applied. After the baseline assessments, a lipid replenishing topical product was applied to the test site, while water was applied to the control site under supervision of Investigator. The test product was applied in consistent quantity ensuring the uniform distribution over the facial site.

Post-application measurements were made at T-15 minutes, allowing evaluation of early instrumental responsiveness and sensitivity to lipid replenishment-associated changes.

Intervention details

The intervention involved a single topical application during the study visit. In this split-face design, the allocation of the test product and control (water) to the left or right facial site was determined using a predefined randomization schedule. The test product was applied topically to the assigned facial site using a standardized procedure, ensuring uniform distribution over the designated assessment area. Application was performed under investigator supervision using a fixed amount of the product, which was gently and evenly spread across the facial site. The control site received a topical application of water using the same method. No further applications were performed, and all assessments were conducted following a single application to evaluate the immediate response. 

The test product used in this study was a commercially available moisturizing lotion, CeraVe® Moisturizing Lotion (New York City, New York), selected as a formulation to induce measurable changes in skin hydration and barrier-related parameters for methodological validation purposes (Table 1).

**Table 1:** Details of test product.

Parameter	Description
Product name	CeraVe® Moisturizing Lotion (New York City, New York)
Product type	Moisturizing lotion for dry to normal skin
Key ingredients	Ceramides, hyaluronic acid, and Glycerine
Manufacturer	L'Oréal (Clichy, France, under the CeraVe brand)
Marketed By	CeraVe (New York City, New York), a brand developed by dermatologists

Instrumental assessments

All instrumental evaluations were conducted by trained assessors who were blinded to product application site assignment to reduce evaluation bias. The following non-invasive instruments were employed:

Skin Glossymeter GL 200 (Cologne, Germany): This was used to assess skin surface gloss and measure the LRF by measuring both directly reflected and diffusely scattered light.

Corneometer® CM 825 (Cologne, Germany): This was used to assess stratum corneum hydration based on capacitance measurement [5].

Tewameter® TM Hex (Cologne, Germany): This was used to assess TEWL as a measurement of skin barrier function [6].

VisioScan® VC 20 Plus (Cologne, Germany): This was used to assess skin surface microrelief parameters, such as smoothness, roughness, and scaliness.

Sebumeter® SM 825 (Cologne, Germany): This was used to quantify skin surface sebum levels using grease spot photometry.

All measurements were conducted at baseline and at T-15 minutes post-application on both facial sites.

The participants were closely observed throughout the study for any adverse events or discomfort. Expected transient reactions such as dryness, mild erythema, tingling sensation, or irritation were recorded. No invasive procedures were conducted.

Environmental control and acclimatization

All evaluation parameters were carried out under controlled environmental conditions to minimize variability in instrumental readings. On the day of evaluation, room temperature and relative humidity were controlled and recorded before testing. The average environmental conditions during assessments were 24.6°C temperature and 40% relative humidity, respectively.

Prior to baseline assessment, participants underwent an acclimatization period of at least 15 minutes in the controlled evaluation room. During this period, participants were asked to remain seated and at rest to allow stabilization of cutaneous blood flow and skin surface conditions.

Safety monitoring and adverse event assessment

Safety was also observed throughout the study by evaluating the participants for presence of any signs of cutaneous intolerance or adverse events at both the test and control sites. The clinical assessments were done by observing and evaluating the participants self-assessment of erythema (redness), burning or stinging sensation, peeling or scaling, itching, dryness, and any other discomfort caused by the application of the product. All observations were made at baseline and during post-application evaluations. Any adverse events, whether expected or unexpected, were documented with respect to onset, severity, duration, and resolution. Participants were instructed to report any delayed reactions, and appropriate medical evaluation was to be provided if required.

Statistical analysis

All statistical analysis was conducted using IBM SPSS Statistics for Windows, Version 29.0.1.0 or higher (Released 2021; IBM Corp., Armonk, New York). Descriptive statistics, such as mean, standard deviation, median, minimum, and maximum, were used to describe continuous variables. Changes from baseline and percentage changes were calculated for post-application measurements.

Participants who withdrew from the study, if any, were not included in the final analysis. Since the study was exploratory in nature, hypothesis testing was not performed.

Sample size determination

Skin parameters including skin shine, sebum levels, TEWL, and skin hydration collectively contribute to the assessment of skin lipid replenishment and reflection characteristics. Among these, skin hydration was selected as the key parameter for sample size determination, as it is a well-established and quantifiable indicator of lipid replenishment and skin barrier function. Accordingly, the sample size calculation was based on the primary endpoint of change in skin hydration (%) from baseline to post-application of the test product. Reference values for mean skin hydration and standard deviation were obtained from previously published studies with similar designs, where mean (± SD) skin hydration increased from 35.0% (± 15.04) at baseline to 46.8% (± 16.7) post-application. Using a paired comparison approach with a one-sided significance level of 5% and a statistical power of approximately 85% to detect a true mean difference, the standardized effect size was calculated to be 0.7346. Based on this, the required sample size was estimated to be 14.78 participants, which was rounded up to 15 participants for study completion.

## Results

Demographics and other baseline characteristics

The study comprised a total of 15 (100%) participants, including 14 (93.33%) women and one man (6.67%). The mean age of the participants was 23.73 ± 2.49 years, the mean weight was 58.22 ± 8.74 kg, and the mean height was 155.79 ± 5.78 cm (Table 2).

**Table 2:** Demographic representation. Demographics: Summary of baseline demographic characteristics of the study participants, including age and gender distribution.

Demographic details for enrolled participants			
Parameter	Statistics	Total enrolled participants, N = 15	Total completed participants, N = 15
Gender M/F/TG	Female	14 (93.33%)	14 (93.33%)
	Male	1 (6.67%)	1 (6.67%)
Predominant race	Asian	15 (100%)	15 (100%)
Medical history (Yes/No)	Yes	-	-
	No	15 (100%)	15 (100%)
Age (years)	N	15	15
	Mean	23.73	23.73
	SD	2.49	2.49
	Median	23	23
	Minimum	18	18
	Maximum	28	28
Weight (kg)	N	15	15
	Mean	58.22	58.22
	SD	8.74	8.74
	Median	57.38	57.38
	Minimum	45.62	45.62
	Maximum	80.45	80.45
Height (cm)	N	15	15
	Mean	155.79	155.79
	SD	5.78	5.78
	Median	153.6	153.6
	Minimum	148.4	148.4
	Maximum	165.2	165.2

Subject Disposition flow chart

**Figure 1:**
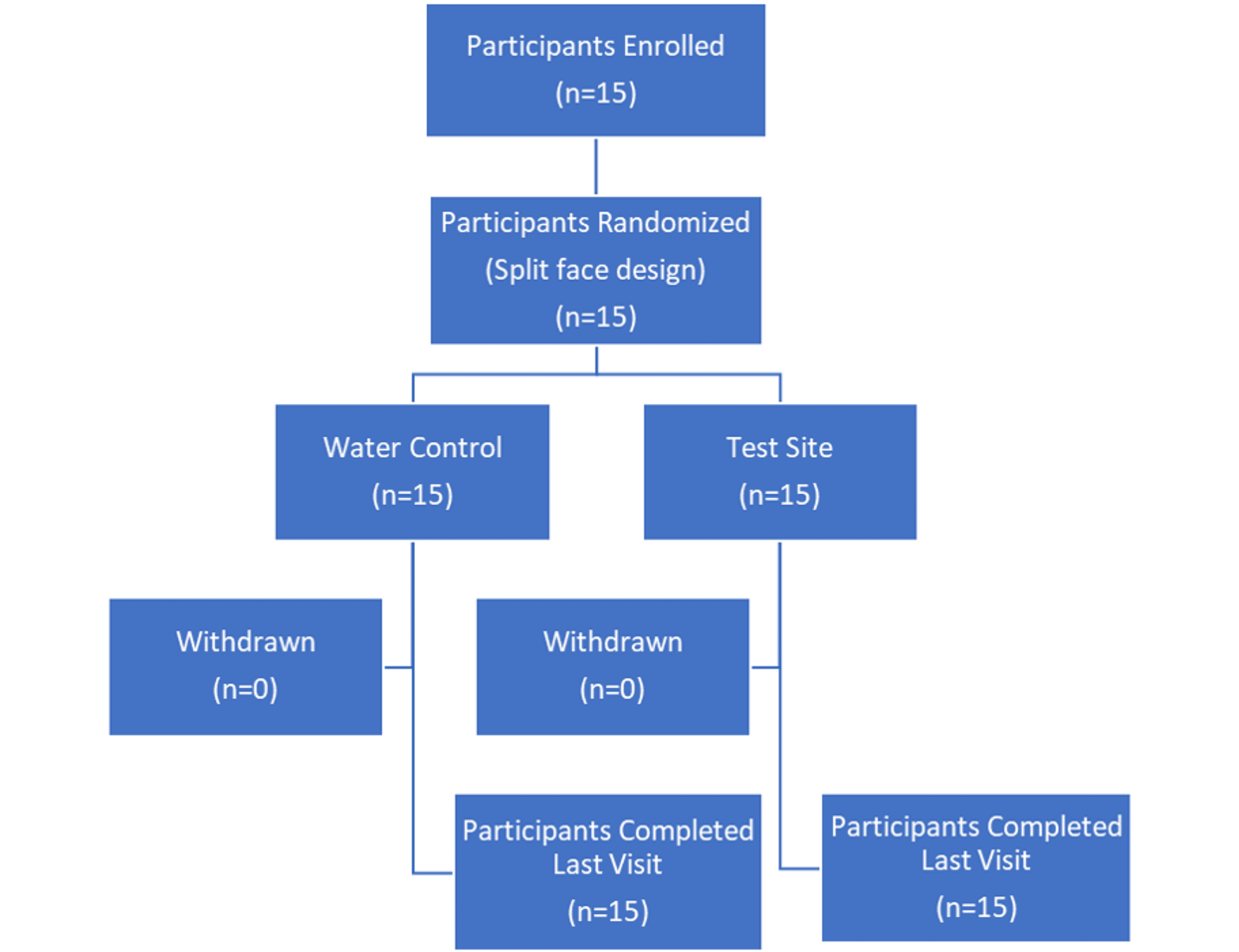
Participants disposition. Disposition: Summary of participant enrollment, allocation, and completion status throughout the study.

A total of 15 participants (100%) were screened and enrolled in the study, and all enrolled participants completed the study as per the study plan. No participant withdrawals, protocol deviations, or discontinuations were reported during the study period (Figure 1).

**Figure 2:**
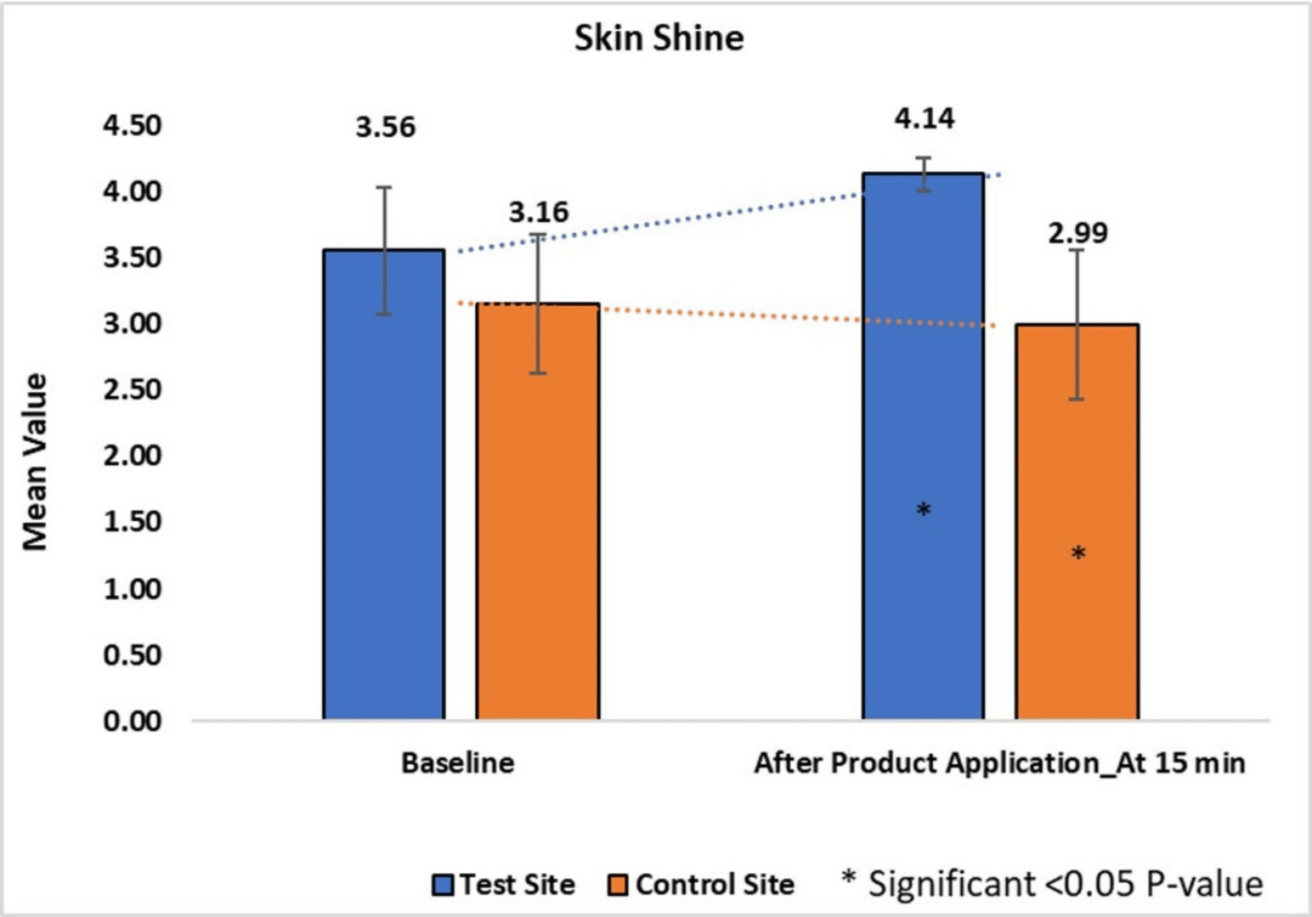
Change in skin shine using Glossymeter® GL 200 (Cologne, Germany). Skin shine values at baseline and post-application at both test and control sites were measured using the Glossymeter® GL 200 (Cologne, Germany). Data are expressed as the mean. A paired t-test was used for statistical analysis, and results are presented with corresponding t values and p values. Statistical significance was set at p < 0.05.

Skin shine: Skin shine assessment showed a measurable improvement at the test site following application of the test product. Mean skin shine values increased from 3.56 ± 0.48 at baseline to 4.14 ± 0.12 post-application with a paired t-test (t value = 4.70), indicating surface reflectance. This change was statistically significant with p value <0.05. In contrast, the control site showed a slight reduction in skin shine, with the mean values decreasing from 3.16 ± 0.52 at baseline to 2.99 ± 0.56 post-application with a paired t-test (t value = -2.99). This change was statistically significant with p value <0.05. These findings put forward a site-specific enhancement in skin shine at the test area, while the control site showed a slight decline (Figure 2).

Skin hydration: Skin hydration assessment showed a marked increase at the test site following application of the test product. Mean hydration values increased from 27.43 ± 3.67 at baseline to 46.20 ± 5.87 post-application with a paired t-test (t value = 15.33), indicating real improvement in hydration. This change was statistically significant with p value <0.05. In contrast, hydration levels at the control site showed a slight decline, decreasing from 33.02 ± 1.39 at baseline to 32.38 ± 1.25 post-application with a paired t-test (t value = -4.26). This change was statistically significant with p value <0.05. These results showed a clear site-specific increase in skin hydration at the test area, whereas hydration at the control site remained relatively constant (Figure 4A)

**Figure 3:**
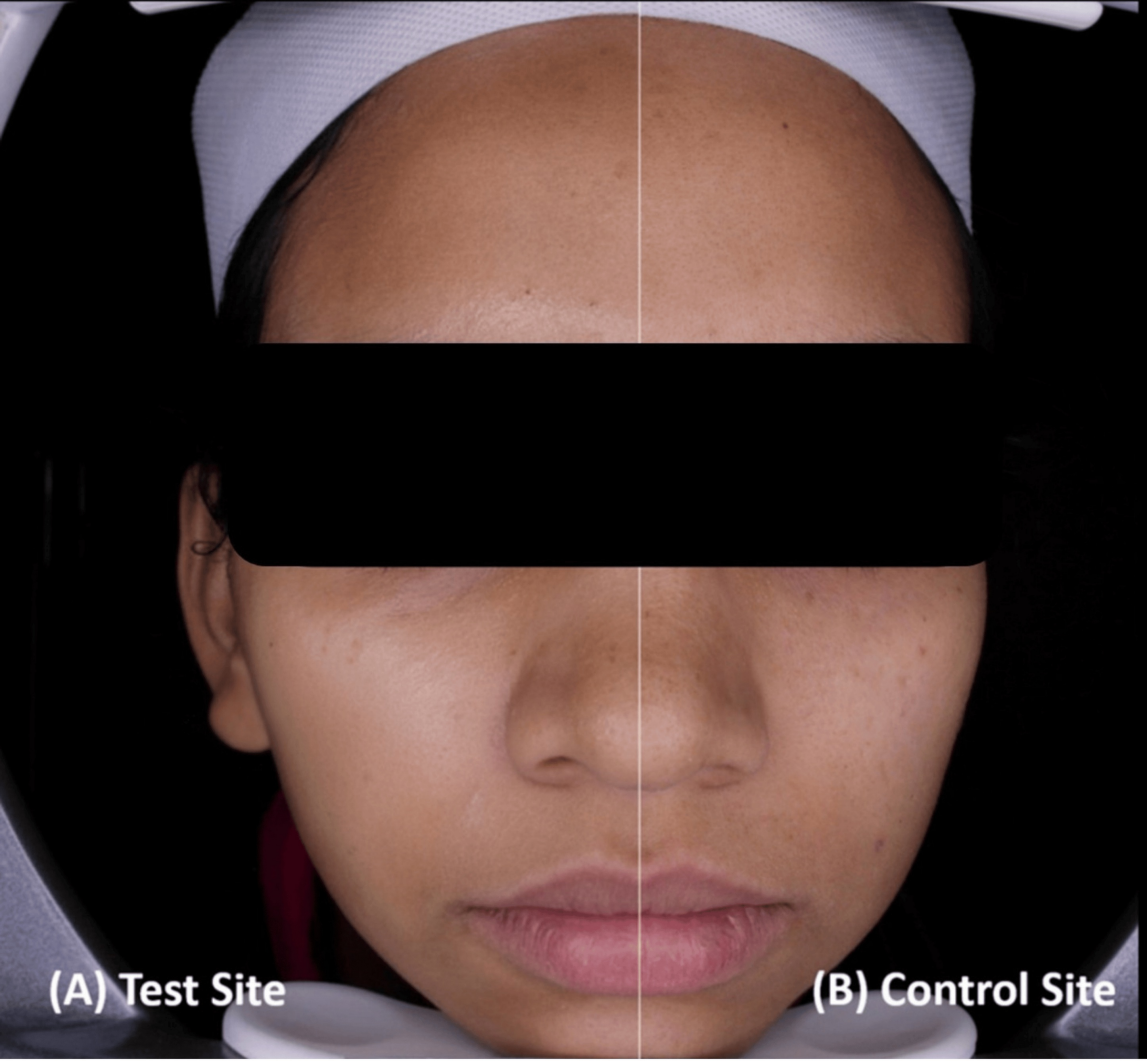
Skin shine using Skin Glossymeter® GL 200 (Cologne, Germany). Skin shine in the test site and control site after application of the test product.

**Figure 4:**
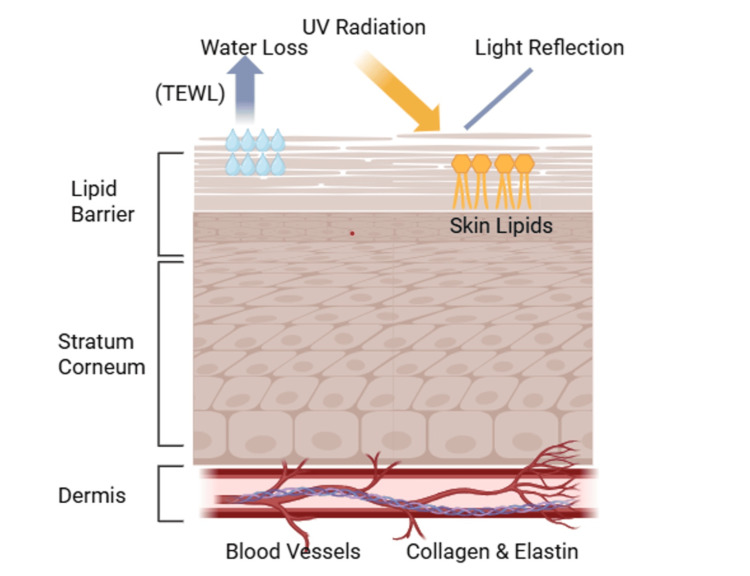
Skin structure and barrier function illustrations. Skin structure: Schematic representation of skin layers, including the stratum corneum, epidermis, and dermis, highlighting key aspects of skin barrier function such as lipid organization, TEWL, and surface light reflection. Created by the authors using BioRender.com (Toronto, Canada). TEWL: transepidermal water loss.

TEWL: TEWL assessment indicated a notable reduction at the test site following application of the test product. Mean values decreased from 15.73 ± 0.60 at baseline to 8.26 ± 0.63 post-application, with a paired t-test (t value = -30.36) demonstrating improved barrier function. This change was statistically significant with p value <0.05. In contrast, the control site showed a slight increase in TEWL, with mean values increasing from 14.59 ± 0.60 at baseline to 15.08 ± 0.72 post-application, with a paired t-test (t value = 3.62). This change was statistically significant with p value <0.05. These findings indicate a site-specific reduction in water loss at the test area, while the control site exhibited a slight increase over the same defined period (Figures 3 and 4B).

**Figure 5:**
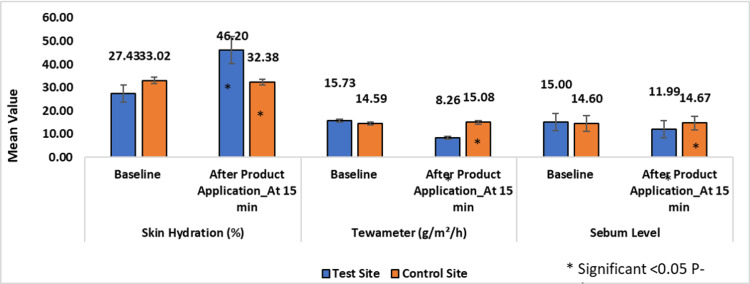
Change in (A) skin hydration using Corneometer® CM 825 (Cologne, Germany); (B) skin TEWL using Tewameter® TM Hex (Cologne, Germany); (C) skin sebum level using Sebumeter® SM 825 (Cologne, Germany). Changes in (A) skin hydration, (B) TEWL, and (C) skin sebum levels measured at baseline and post-application at both test and control sites. Data are expressed as the mean. A paired t-test was used for statistical analysis, and results are presented with corresponding t values and p values. Statistical significance was set at p < 0.05. TEWL: transepidermal water loss.

Sebum level: Sebum level assessment demonstrated a great reduction at the test site following application of the test product. Mean sebum values decreased from 15.00 ± 3.69 at baseline to 11.99 ± 3.68 post-application, with a paired t-test (t value = -3.92), indicating a reduction in skin sebum levels. This change was statistically significant with p value <0.05. At the control site, sebum levels remained stable, with values showing minimal change from 14.60 ± 3.33 at baseline to 14.67 ± 2.80 post-application, with a paired t-test (t value = 0.17). This change was not statistically significant with p value >0.05. These results indicate that sebum levels at the test site decreased following application, while the control site showed no meaningful variation over the defined period (Figure 5C).

**Figure 6:**
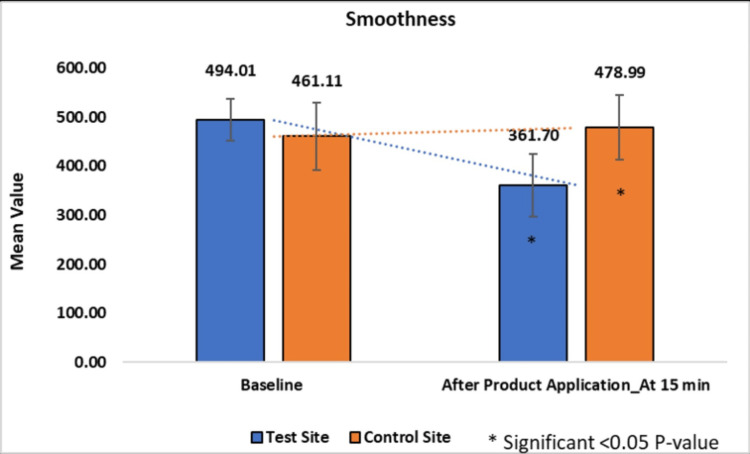
Change in skin smoothness using VisioScan® VC 20 Plus (Cologne, Germany). Skin smoothness values measured at baseline and post-application at both test and control sites using the VisioScan® VC 20 Plus (Cologne, Germany). Data are expressed as the mean. A paired t-test was used for statistical analysis, and results are presented with corresponding t values and p values. Statistical significance was set at p < 0.05.

**Figure 7:**
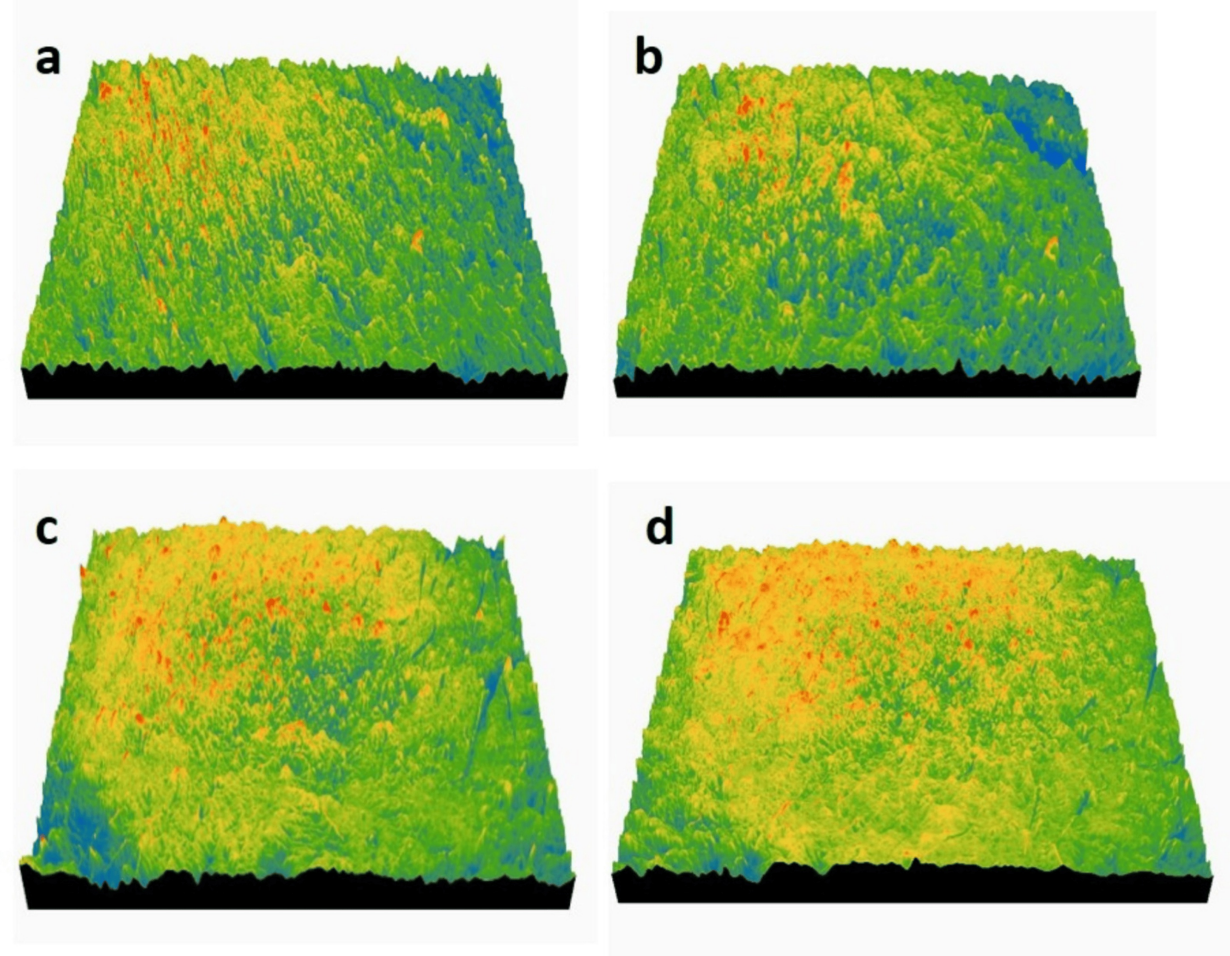
Change in 3D topographical image of skin smoothness before and after application. (A) Change in the 3D topographical image of skin smoothness before the control application. (B) Change in the 3D topographical image of skin smoothness after the control application. (C) Change in the 3D topographical image of skin smoothness before the test product application. (D) Change in the 3D topographical image of skin smoothness after the test product application.

Skin Smoothness: Skin smoothness was evaluated where lower values indicate improved smoothness. At the test site, skin smoothness values decreased from 494.01 ± 42.35 at baseline to 361.70 ± 63.99 post-application, with a paired t-test (t value = -7.16), reflecting an improvement in skin smoothness following application of the test product. This change was statistically significant with p value <0.05. In contrast, the control site demonstrated an increase in smoothness values, rising from 461.11 ± 69.19 at baseline to 478.99 ± 65.25 post-application, with a paired t-test (t value = 2.72), indicating a reduction in skin smoothness. This change was statistically significant with p value <0.05. These findings showed a site-specific improvement in skin smoothness at the test area, while the control site exhibited a deterioration over the same defined period (Figures 6 and 7).

**Figure 8:**
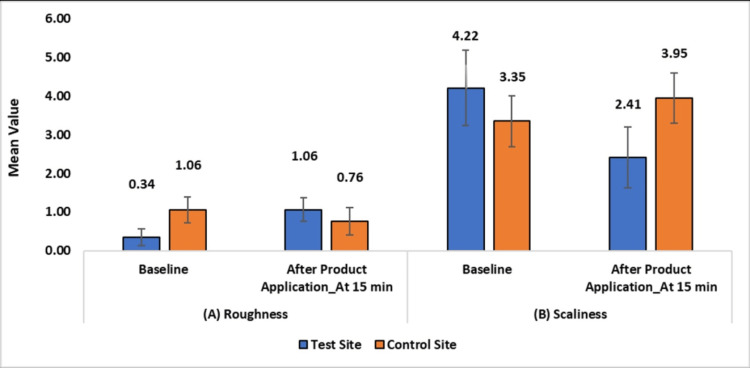
Change in (A) skin roughness using VisioScan® VC 20 Plus (Cologne, Germany); (B) skin scaliness using VisioScan® VC 20 Plus (Cologne, Germany). Changes in (A) skin roughness and (B) skin scaliness measured at baseline and post-application at both test and control sites. Data are expressed as the mean. A paired t-test was used for statistical analysis, and results are presented with corresponding t values and p values. Statistical significance was set at p < 0.05.

Skin roughness: Skin roughness was assessed where higher values indicate reduced skin roughness. At the test site, skin roughness values increased from 0.34 ± 0.22 at baseline to 1.06 ± 0.31 post-application, with a paired t-test (t value = 8.70), indicating a reduction in skin roughness following application of the test product. This change was statistically significant with p value <0.05. In contrast, the control site showed a decrease in roughness values from 1.06 ± 0.34 at baseline to 0.76 ± 0.35 post-application, with a paired t-test (t value = -5.01) reflecting an increase in skin roughness. This change was statistically significant with p value <0.05. These findings demonstrate a site-specific reduction in skin roughness at the test area, while the control site exhibited a deterioration over the same assessment period (Figure 8A).

Skin scaliness: Skin scaliness assessment showed a reduction at the test site following application of the test product. Mean scaliness values decreased from 4.22 ± 0.97 at baseline to 2.41 ± 0.79 post-application, with a paired t-test (t value = -6.86), indicating an improvement in skin surface condition. This change was statistically significant with p value <0.05. In contrast, the control site exhibited an increase in scaliness, with mean values rising from 3.35 ± 0.65 at baseline to 3.95 ± 0.65 post-application, with a paired t-test (t value = 4.29). This change was statistically significant with p value <0.05. These results indicate a site-specific reduction in skin scaliness at the test area, while the control site showed a worsening of scaliness over the same defined period (Figure 8B).

## Discussion

The present study was conducted as a methodological standardization and validation study to evaluate the ability of a structured skin assessment methodology to detect changes associated with lipid replenishment and the LRF using integrated instrumental evaluations. The results showed that the standardized assessment framework was capable of detecting localized changes in skin barrier function and surface morphology while maintaining physiological stability at the control sites. These findings support the reliability and responsiveness of the methodological framework under controlled experimental conditions.

Within the methodological framework, skin surface properties were evaluated through parameters associated with the LRF. The LRF concept is governed by two principal measurable components: skin shine and skin sebum levels, which together influence the reflective behavior of the skin surface. Skin shine represents the degree of direct light reflection from the skin surface, while sebum levels contribute to the lipid-related skin-reflecting characteristics that influence surface reflectance. In the present study, skin shine demonstrated a small increase at the test site following application, whereas the control site remained relatively stable. Importantly, sebum levels showed a reduction at the test site and remained stable at the control site. This observation suggests that the improvement in skin appearance was not only driven by excess surface oil but also by improved surface organization, including smoothness, roughness, and scaliness of the skin. Such findings are particularly relevant for methodological validation, as they confirm that the measurements respond to true surface structural changes rather than superficially. Previous studies have demonstrated that clinical changes in skin reflectance are closely linked to skin microtopography and barrier integrity, rather than temporary surface artifacts or cosmetic film effects [16,17].

In addition to skin parameters, the methodological framework evaluated lipid replenishment-related skin barrier parameters, which were measured through skin hydration, skin shine, TEWL, and surface microrelief characteristics, including skin roughness, skin smoothness, and scaliness. An increase in skin hydration accompanied by a reduction in TEWL was observed at the test site, while control sites remained largely unchanged. These coordinated responses are consistent with the established understanding that restoration of the intercellular lipid matrix enhances stratum corneum barrier integrity, increases water-holding capacity, and reduces passive water diffusion across the epidermis [18].

Surface parameters provided additional evidence supporting lipid replenishment-associated structural improvements. The study demonstrated improvements in skin smoothness together with reductions in skin roughness and scaliness at the test site following product application. These changes reflect improved corneocyte cohesion and reduced surface irregularities, which occur when the lipid organization of the stratum corneum is restored. Such structural improvements are known to influence both the functional barrier properties and the reflecting appearance of the skin surface [19].

Importantly, sebum levels remained within normal physiological ranges, indicating that the observed improvements were not driven by excess surface oil but rather by restoration of structured lipid balance. This distinction has been emphasized in prior bioengineering studies evaluating barrier repair and moisturization.

A central methodological focus of this study was the evaluation of the LRF as an integrated indicator of lipid-replenishment-associated surface changes. LRF reflects the balance between directly reflected and diffusely scattered light, which is influenced by surface smoothness, hydration, and lipid-mediated organization of the stratum corneum. Previous investigations into skin optics have demonstrated strong associations between surface roughness, hydration, and reflectance behavior, supporting the scientific rationale for composite skin reflecting parameters. The alignment of LRF behavior with improvements in hydration and surface smoothness observed in this study supports its potential utility as a holistic marker of lipid-associated surface refinement.

The integration of skin reflecting and biophysical measurements represents an important methodological advancement in dermatological and cosmetic research. Previous studies have demonstrated the value of combining multiple instrumental parameters to provide a comprehensive evaluation of skin barrier function and surface morphology [7]. In the present study, the combined evaluation of LRF-related parameters (skin shine and sebum) together with lipid replenishment-related biophysical parameters (hydration, TEWL, smoothness, roughness, and scaliness) enabled a more comprehensive assessment of skin surface and barrier-related changes. This multidimensional methodological approach strengthens the interpretation of instrumental findings and enhances the robustness of skin assessment methodologies.

International guidelines further support the methodological framework adopted in the present study. The European Expert Group on Efficacy Measurement of Cosmetics and Other Topical Products (EEMCO) recommends standardized acclimatization, controlled environmental conditions, and intra-subject comparison designs for the assessment of moisturizers and barrier-related outcomes [20,21]. The current study adhered closely to these recommendations, thereby strengthening the validity and reproducibility of the instrumental findings.

In previous work, the imaging approach was standardized and validated to evaluate the immediate mattifying effects of topical skin care products, primarily focusing on surface changes. That study demonstrated the feasibility of using imaging-based techniques for objective assessment of rapid surface modifications following product application. Building upon this methodological foundation, the present investigation expands the assessment framework by incorporating additional complementary biophysical parameters, including skin hydration, TEWL, surface microrelief (smoothness, roughness, and scaliness), and sebum levels. The integration of these instrumental measures alongside reflection analysis enables a more comprehensive evaluation of lipid replenishment-associated changes, capturing not only surface appearance but also underlying barrier-related functional improvements. This multidimensional approach strengthens the interpretability of findings and enhances the robustness of non-invasive skin assessment methodologies [22].

The findings of this study should be interpreted in the context of its exploratory design. The short, post-application assessment window was intentionally selected to evaluate immediate instrument responsiveness rather than long-term biological adaptation. While this approach is appropriate for validation purposes, longer-duration studies may be required to fully characterize the behavior of the LRF and related parameters during sustained lipid replenishment. Additionally, the modest sample size reflects the methodological focus of the study and is consistent with commonly accepted practices in skin instrumentation validation research.

Overall, this study establishes a standardized and reproducible framework for assessing lipid replenishment-associated changes using integrated methodology. The results demonstrate that the combined assessment approach is sensitive to early changes in skin barrier function and surface morphology while maintaining measurement stability under controlled experimental conditions. Furthermore, the findings support the potential role of the LRF as an integrated skin reflectance parameter governed by skin shine and sebum levels, while lipid replenishment-related changes can be effectively evaluated through hydration, TEWL, and surface microrelief parameters including roughness and smoothness. This validated methodological framework may serve as a valuable foundation for future dermatological and cosmetic research focused on skin barrier restoration and surface refinement.

This study has several limitations that should be considered when interpreting the findings. The study employed a short post-application assessment window, which was intentionally selected to evaluate the immediate performance and responsiveness of the standardized assessment methodology; however, this reflects only short-term responses rather than long-term effects. The sample size was relatively small (n = 15), consistent with the exploratory and methodological validation design, which may limit generalizability. Additionally, the study population was relatively homogeneous in terms of age group and ethnicity, potentially restricting the broader applicability of the findings. While these design elements are appropriate for validation purposes and align with commonly accepted practices in instrumentation-based studies, future investigations with larger, more diverse populations and extended follow-up durations are warranted to further validate these observations and to explore the temporal behavior of the evaluated parameters, including the LRF.

## Conclusions

This study establishes a standardized and validated methodology for assessing skin changes associated with lipid replenishment using integrated skin reflectance and biophysical measurements. The applied methodology demonstrated the ability to detect rapid, site-specific changes in key barrier-related parameters, including skin hydration, TEWL, and skin surface microrelief characteristics such as smoothness, roughness, and scaliness, while maintaining physiological stability at the control sites. These findings support the reliability of the standardized measurement approach under controlled experimental conditions.

Within this framework, the evaluation of LRF was performed through parameters related to skin shine and sebum levels, which together influence the reflective properties of the skin surface. The results indicate that LRF has the potential to function as an integrated indicator reflecting skin surface refinement associated with lipid replenishment. At the same time, lipid replenishment-related changes were effectively characterized through complementary biophysical parameters, including skin hydration, TEWL, and skin surface microrelief parameters such as roughness and smoothness. Overall, the validated methodological framework demonstrates the value of combining optical and biophysical measurements to capture both surface appearance- and barrier-related functional changes. This standardized assessment approach may serve as a useful foundation for future dermatological and cosmetic research investigating skin barrier restoration, lipid replenishment, and improvements in skin surface quality.
